# Associations of resistance liuzijue qigong with sleep and mood in chronic insomnia: a pilot open-label Non-randomized trial

**DOI:** 10.3389/fmed.2026.1903577

**Published:** 2026-09-08

**Authors:** Chenglong Hu, Huihui Huang, Mengdi Zhou, Qiantong He, Aisong Guo

**Affiliations:** 1 Affiliated Hospital of Nantong University, Nantong, Jiangsu, China; 2 School of Nursing and Rehabilitation, Nantong University, Nantong, China; 3 The Fourth People’s Hospital of Nantong City, Nantong, Jiangsu, China

**Keywords:** chronic insomnia, emotional status, non-pharmacological intervention, resistance liuzijue qigong, sleep parameters

## Abstract

**Background:**

Chronic insomnia is highly prevalent worldwide. First-line treatments are constrained by adverse effects, dependence, and limited accessibility. Traditional mind-body exercises such as Resistance Liuzijue Qigong are safe and feasible. This prospective pilot study preliminarily explored associations between Qigong practice and changes in sleep, mood, fatigue, and quality of life in patients with chronic insomnia.

**Methods:**

In this prospective open-label non-randomized pilot trial, 40 patients with chronic insomnia were assigned to intervention or control groups. The intervention group underwent 3 months of Resistance Liuzijue Qigong training, while the control group received routine health education. Primary outcomes were the Pittsburgh Sleep Quality Index (PSQI) and actigraphy-derived objective sleep parameters; secondary outcomes included the Self-Rating Anxiety Scale (SAS), Self-Rating Depression Scale (SDS), Fatigue Severity Scale (FSS), 36-Item Short Form Health Survey (SF-36), and nocturnal mean heart and respiratory rates. Normally distributed data were analyzed with paired and independent t-tests; skewed data with paired rank-sum and Mann-Whitney U tests; categorical data with chi-square or Fisher's exact test. Significance was set at *α* = 0.05.

**Results:**

Comparisons between the 3-month Resistance Liuzijue Qigong intervention group and the routine care control group showed significant within-group reductions in PSQI, SAS, SDS and FSS scores for both groups (all *P*<0.001). Between-group comparisons revealed numerically lower SAS (*t*=4.268, *P*<0.001) and SDS (*t*=2.371, *P*=0.024) scores in the intervention group compared to the control group. For objective sleep parameters, the between-group comparison showed a statistically significant difference in sleep efficiency (*t*=−3.049, *P*=0.004) and wake after sleep onset (*U*=284.5, *P*=0.023). The between-group difference in the change rate of sleep efficiency was also statistically significant (*W*=284, *P*=0.023).

**Conclusions:**

This pilot study suggested that Resistance Liuzijue Qigong may be associated with anxiety, depression, objective sleep continuity, and sleep efficiency in chronic insomnia patients. However, given the non-randomized open-label design, these findings are exploratory and require confirmation in large-scale RCTs with allocation concealment.

## 1 Introduction

Chronic Insomnia (CI) is defined as persistent difficulty in falling or staying asleep, or non-restorative sleep accompanied by daytime dysfunction, despite adequate sleep opportunities. According to the diagnostic criteria, the symptoms occur at least three times per week and last for no less than three months to confirm the diagnosis (1). Epidemiological investigations demonstrate that the global prevalence of insomnia ranges from 10% to 20%, and nearly half of insomnia patients eventually develop chronic insomnia (2). CI is not merely a single sleep disorder; it forms a bidirectional pathological correlation with psychiatric disorders, cardio-cerebrovascular diseases, metabolic syndrome and cognitive decline. Insomnia exacerbates physical and mental impairments, and these comorbidities in turn disrupt sleep architecture, creating a vicious cycle and greatly increasing the difficulty of clinical treatment (3).

Currently, sedative-hypnotic medications and cognitive behavioral therapy for insomnia (CBT-I) are recognized as the two first-line interventions for CI (4). Pharmacotherapy can rapidly relieve sleep disturbances, but long-term use is associated with adverse reactions such as daytime drowsiness and dizziness, as well as drug dependence, drug tolerance and post-withdrawal symptom rebound. Although CBT-I achieves sustained therapeutic effects without drug-related side effects, it requires professional therapists, specific venues and high patient compliance, which limits its large-scale application in primary medical institutions and general populations (5). Therefore, exploring safe, non-invasive, simple and widely applicable non-pharmacological interventions has become a research hotspot in the field of chronic insomnia.

Liuzijue Qigong is a traditional Chinese breathing and mind-body exercise that integrates vocal breathing, rhythmic respiration and physical movements to harmonize physical and mental states. It can simultaneously regulate body posture, respiration and concentration. Accumulated studies have verified that Liuzijue Qigong modulates autonomic nervous function by increasing vagal tone, thereby reducing heart rate and blood pressure and relieving physical stress responses (6). In addition, slow breathing combined with vocalization can activate the dorsal anterior cingulate cortex-pontine caudate nucleus (dACC-PnC) neural pathway. This pathway regulates the respiratory centers in the pons and medulla oblongata as well as emotion-related brain regions, reduces respiratory rate and alleviates anxiety, and ultimately improves emotional status and sleep quality (7). Resistance Liuzijue Qigong is a novel mind-body exercise developed by integrating traditional Liuzijue Qigong with modern exercise anatomy and resistance training theories. It organically combines traditional breathing guidance, mindfulness relaxation and standardized resistance training. Previous studies have confirmed that weight-bearing Liuzijue Qigong can effectively improve cardiopulmonary function and muscle strength, as well as exercise endurance, showing good application prospects in cardiopulmonary rehabilitation (8, 9). Compared with traditional Liuzijue Qigong, the addition of resistance loads enhances physical stimulation and metabolic regulation, which theoretically produces synergistic effects with respiratory and neural modulation to achieve better outcomes for sleep and emotional disorders. Nevertheless, targeted clinical studies focusing on the efficacy and safety of Resistance Liuzijue Qigong in patients with chronic insomnia remain absent, and relevant clinical evidence is still insufficient.

Therefore, this prospective controlled pilot study enrolled patients with chronic insomnia to descriptively observe the changes in subjective sleep scores, objective sleep parameters, emotional status, fatigue, and quality of life before and after Resistance Liuzijue Qigong intervention. The present study aimed to explore preliminary associations of this exercise with clinical outcomes, generate hypotheses for future research, and provide pilot data for the non-pharmacological intervention system for this disease.

## 2 Materials and methods

### 2.1 Participant selection

This prospective, open-label, non-randomized pilot controlled study was carried out in the outpatient and inpatient departments of the Department of Traditional Chinese Medicine, the Affiliated Hospital of Nantong University, from March to May 2026. Both inter-group comparison and intra-group pre-post comparison were adopted for data analysis. Participants in the intervention group were patients with chronic insomnia who planned to receive Resistance Liuzijue Qigong training in our ward. The control group was selected from concurrent outpatients and inpatients via systematic sampling based on medical record numbers.

In the recruitment design, we attempted to balance basic demographic and disease-related baseline characteristics by enrolling subjects within the same time window and applying unified inclusion and exclusion criteria for both groups. The matching idea during recruitment was to select control patients with consistent age range, chronic insomnia duration and comorbidity status comparable to intervention participants, so as to minimize baseline differences caused by disparate recruitment channels.

**Figure 1:**
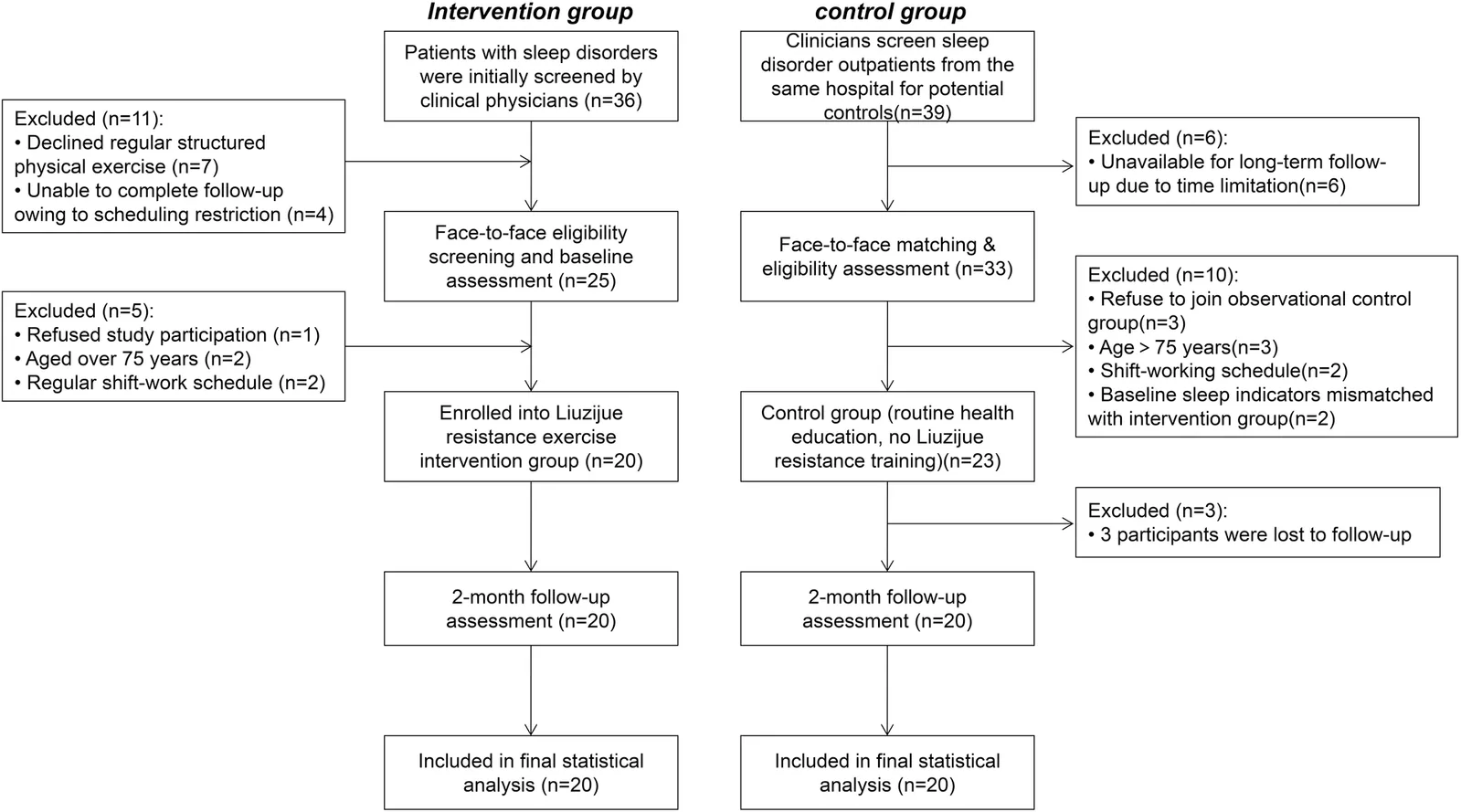
Flow diagram of participant screening, enrollment, exclusion and follow-up in patients with chronic insomnia.

Given the exploratory pilot nature of this study, the sample size was not calculated based on formal power analysis but was determined according to practical recruitment feasibility and the general convention for pilot studies. A total of 36 candidates were initially screened for the intervention group; after multiple eligibility assessments and loss to follow-up, 20 participants were finally included in the statistical analysis. For the control group, 39 potential participants were initially screened, and 20 eligible subjects were enrolled after unified inclusion criteria and preliminary baseline matching. Detailed information on participant exclusion is shown in Figure 1.

### 2.2 Eligibility criteria

#### 2.2.1 Inclusion criteria

(1) Met the diagnostic criteria for chronic insomnia;
(2) Aged between 18 and 75 years old;
(3) Able to understand questionnaire scales and actively cooperate with intervention and assessments;
(4) With normal physical mobility;
(5) Voluntarily participated in this study and signed the informed consent form.

#### 2.2.2 Exclusion criteria

(1) Suffered from uncontrolled severe underlying diseases, including persistent pain, malignancies, cardio-cerebrovascular diseases (hypertension, coronary heart disease, cerebral infarction, craniocerebral injury, Parkinson's disease, dementia, epilepsy, etc.), endocrine disorders (diabetes, hyperthyroidism, hypothyroidism, etc.), neuromuscular diseases and gastroesophageal reflux disease, all of which may induce insomnia;
(2) Pregnant or lactating women;
(3) Shift workers;
(4) Participants enrolled in other concurrent clinical trials.

### 2.3 Intervention protocol

All participants in the two groups received routine health education and basic nursing provided by the Department of Traditional Chinese Medicine. The intervention group additionally received Resistance Liuzijue Qigong training, while no exercise intervention was implemented in the control group.

Certified rehabilitation therapists provided one-on-one on-site training for all participants in accordance with *Liuzijue Qigong Standard Manual* (People's Sports Publishing House, 2021). The therapists demonstrated and explained the mouth shape, breathing patterns and key movements of the six postures in detail. Elastic bands were used to apply resistance loads during movements based on the kinesiological and kinetic analysis of Liuzijue Qigong (Supplementary Materials).

After participants mastered the standard movements, official instructional videos released by the General Administration of Sport of China were distributed for home practice. Multiple supporting measures were adopted to improve training adherence: (1) Weekly WeChat reminders of training tasks; (2) Telephone follow-up every two weeks to inquire about training difficulties; (3) Monthly offline centralized check-ins to correct non-standard movements and encourage persistence; (4) Training log books issued to each participant to record daily practice time and completion. One-on-one follow-up supervision was arranged to record training conditions and ensure training frequency and movement standardization. Detailed training parameters were set as follows: each set consisted of six vocal postures, and each posture was repeated six times. The resistance of elastic bands was 10–20 pounds. The perceived exertion of upper limbs was rated 4–5 points on the OMNI perceived exertion scale, equivalent to 40% to 50% of the one-repetition maximum (1RM) (10). Each training session lasted 15 minutes, twice a day, five days per week, for a total of 3 consecutive months.

Training adherence was calculated as the ratio of completed to prescribed training sessions over the 3-month period. Adherence data for all intervention participants were summarized in Supplementary Table S1, and the mean rate was 92.74%.

### 2.4 Outcome measures

All indicators were assessed at baseline (Month 0) and the 3rd month after intervention. Primary outcomes included the Pittsburgh sleep quality index (PSQI) and objective sleep parameters detected by wrist-worn actigraphs, including total sleep time (TST), sleep onset latency (SOL), wake after sleep onset (WASO) and sleep efficiency (SE).

The actigraphy model used in this study was the ActiGraph wGT3X-BT wearable activity monitor. Participants were required to wear the device continuously on their non-dominant wrist for 7 consecutive nights at each assessment time point, with a minimum of 5 valid recording nights retained for aggregate statistical calculation. Sleep-wake scoring was automatically performed via the validated Sadeh algorithm embedded in ActiLife 6.13 software, and visual manual review was conducted by trained researchers to correct misclassified sleep/wake episodes. Nights with less than 4 hours of valid recording data were defined as invalid and excluded from analysis; missing night records were not imputed, and only participants with ≥5 valid nights were included in final parameter averaging. Before formal data collection, all instruments underwent uniform factory calibration and inter-device consistency verification to guarantee detection reliability.

Secondary outcomes contained scores of anxiety, depression, fatigue, quality of life, as well as nocturnal mean heart rate and mean respiratory rate. All subjective evaluations were conducted using validated self-reported scales, which were described as follows:

PSQI. This self-rated scale was used to evaluate comprehensive sleep status over the past 3 months. It contained 19 items divided into 7 domains: subjective sleep quality, sleep onset latency, total sleep time, habitual sleep efficiency, sleep disturbances, use of hypnotic drugs, and daytime dysfunction. Each item was scored from 0 to 3, with a total score ranging from 0 to 21. Higher scores indicated poorer sleep quality (11). The Cronbach's *α* coefficient of PSQI in this study was 0.79.

Self-rating anxiety scale (SAS) and self-rating depression scale (SDS). Both scales consisted of 20 items, with each item scored from 1 to 4 and the total raw score ranging from 20 to 80. The standard score was calculated by multiplying the raw score by 1.25. For SAS, scores of 50–59 indicated mild anxiety, 60–69 indicated moderate anxiety, and scores above 70 indicated severe anxiety. For SDS, scores of 53–62 indicated mild depression, 63–72 indicated moderate depression, and scores above 72 indicated severe depression. Higher scores reflected more severe anxiety and depressive symptoms (12). The Cronbach's *α* coefficients were 0.82 for SAS and 0.83 for SDS in this study.

Fatigue severity scale (FSS). This scale was applied to assess the severity, frequency and daily interference of fatigue. It included 9 items, and the total score was the sum of all item scores. A total score less than 36 suggested no obvious fatigue, while a score of 36 or above indicated the presence of fatigue requiring further intervention (13). The Cronbach's *α* coefficient of FSS in this study was 0.77.

36-Item Short Form Health Survey (SF-36). This scale comprised 36 items and 8 domains, including physical functioning, role limitations due to physical health, bodily pain, general health, vitality, social functioning, role limitations due to emotional problems and mental health. It was suitable for evaluating health status and quality of life across all age groups and disease populations. The total score was calculated by summing scores of each domain, and the scale was sensitive to changes after intervention (14). The Cronbach's *α* coefficient of SF-36 in this study was 0.79.

### 2.5 Statistical analysis

All statistical analyses were performed using R 4.2.3 software, with the significance level set at *α* = 0.05. The Shapiro–Wilk test was used for normality testing (see Supplementary Tables S2–S5). Normally distributed measurement data were presented as mean ± standard ($\bar{x}\pm s$), and skewed measurement data were expressed as median (interquartile range). Categorical data were described as cases (percentage). The Chi-square test was used for inter-group comparison of categorical data, and Fisher's exact test was adopted when theoretical frequencies were insufficient. For normally distributed data, paired t-test was used for intra-group pre-post comparison, and independent t-test for inter-group comparison.

Cohen's d was calculated as standardized effect size for all t-test analyses. For skewed data such as SOL and WASO, paired rank-sum test and Mann–Whitney U test were applied for intra-group and inter-group comparisons, respectively, with Hodges–Lehmann estimators reported to reflect median intergroup shifts. For skewed data such as SOL and WASO, paired rank-sum test and Mann–Whitney U test were applied for intra-group and inter-group comparisons, respectively. The formula for calculating the percentage change of each scale score before and after intervention is: (post-intervention value-baseline value)/baseline value  ×  100.

Post-hoc power analyses for all between-group comparisons of primary and secondary outcomes were further conducted via G*Power 3.1. We calculated the observed statistical power corresponding to the actual effect size of each indicator, as well as the minimum detectable Cohen's d at a target power of 0.8 to quantify the risk of Type II error caused by the limited sample size (Supplementary Table S6). Furthermore, analysis of covariance was used for sensitivity analysis to adjust for variables with significant baseline intergroup differences and reduce confounding bias from unbalanced baseline data.

### 2.6 Ethical approval

This study was reviewed and approved by the Ethics Committee of the Affiliated Hospital of Nantong University (Approval No.: 2023-K019). All participants were fully informed of the study protocol and signed written informed consent prior to enrollment.

## 3 Results

### 3.1 Baseline characteristics of participants

A total of 40 participants were enrolled in this study, with 20 cases in the intervention group and 20 cases in the control group. No significant inter-group differences were found in age, gender, body mass index, educational background, occupation and household registration (all *P* > 0.05). A significant difference in baseline physical activity level was observed between the two groups (*P* < 0.001). In terms of scale scores, the SAS score of the intervention group was significantly higher than that of the control group (*t* = 3.400, *P* = 0.002), while no significant differences were detected in PSQI, SDS, FSS and SF-36 scores (all *P* > 0.05). For objective sleep parameters, significant inter-group differences existed in baseline SE (*t* = −3.190, *P* = 0.003), SOL (*U* = 276, *P* = 0.041) and WASO (*U* = 278, *P* = 0.037). TST, mean heart rate and mean respiratory rate were comparable between the two groups at baseline (Table 1 and Supplementary Table S7).

**Table 1:** Baseline characteristics of enrolled subjects.

Items	Intervention group (*n*=20)	Control group (*n*=20)	Statistical value	*P*
General demographic data				
Age (years, $\bar{x}\pm s$)	52.30 ± 14.19	50.50 ± 12.96	*t*=−0.419	0.678
Gender [n(%)]			*χ^2^*=0.125	0.723
Male	5 (25.0)	6 (30.0)		
Female	15 (75.0)	14 (70.0)		
BMI (kg/m^2^, $\bar{x}\pm s$)	23.38 ± 2.42	22.84 ± 3.11	*t*=−0.618	0.540
Educational level [n(%)]			*χ^2^*=1.072	0.784
Primary school and below	6 (30.0)	4 (20.0)		
Junior high school	7 (35.0)	10 (50.0)		
Senior high school	4 (20.0)	3 (15.0)		
College or above	3 (15.0)	3 (15.0)		
Occupation [n(%)]			*χ^2^*=2.692	0.260
Retired	8 (40.0)	5 (25.0)		
Manual worker/technician/salesperson/farmer	7 (35.0)	5 (25.0)		
Clerk/student/teacher	5 (25.0)	10 (50.0)		
Household registration [n(%)]			*χ^2^*=0.107	0.744
Urban	13 (65.0)	12 (60.0)		
Rural	7 (35.0)	8 (40.0)		
Physical activity level [n(%)]			−	<0.001^a^
Insufficient	0 (0.0)	12 (60.0)		
Moderate	11 (55.0)	4 (20.0)		
Vigorous	9 (45.0)	4 (20.0)		
Baseline sleep and psychological scales				
Pittsburgh Sleep Quality Index ($\bar{x}\pm s$)	13.70 ± 4.75	15.30 ± 2.92	*t*=1.280	0.207
36-Item Short Form Health Survey ($\bar{x}\pm s$)	64.86 ± 15.86	56.98 ± 12.95	*t*=−1.720	0.093
Self-Rating Anxiety Scale ($\bar{x}\pm s$)	49.90 ± 8.52	41.80 ± 6.41	*t*=3.400	0.002
Self-Rating Depression Scale ($\bar{x}\pm s$)	50.25 ± 7.01	45.25 ± 10.33	*t*=1.790	0.081
Fatigue Severity Scale [median (IQR)]	50.50[36.00,57.50]	49.50[47.75,53.00]	*U*=190	0.786^b^
Baseline objective sleep parameters (actigraphy)				
Total Sleep Time (min, $\bar{x}\pm s$)	305.10 ± 88.52	289.45 ± 69.84	*t*=−0.621	0.539
Sleep Efficiency (%, $\bar{x}\pm s$)	0.68 ± 0.16	0.53 ± 0.13	*t*=−3.190	0.003
Sleep Onset Latency [min, median (IQR)]	57.50[24.75,93.25]	106.50[26.25,150.00]	*U*=276	0.041^b^
Wake After Sleep Onset [min, median (IQR)]	45.00[27.25,130.25]	139.00[61.75,203.25]	*U*=278	0.037^b^
Mean heart rate (beats/min, $\bar{x}\pm s$)	62.55 ± 5.54	63.45 ± 8.65	*t*=0.392	0.697
Respiratory rate [breaths/min, median (IQR)]	15[13,16.25]	15[13,15.25]	*U*=156	0.231^b^

Footnote: Normally distributed continuous variables were analyzed using independent-samples t-test; categorical variables were compared with the Pearson *χ^2^* test.

$\bar{x}\pm s$: mean ± standard deviation; median [IQR]: median (interquartile range).

a Fisher’s exact test.

b Mann–Whitney Utest.

### 3.2 Intra-group and inter-group comparisons after 3-month intervention

**Figure 2:**
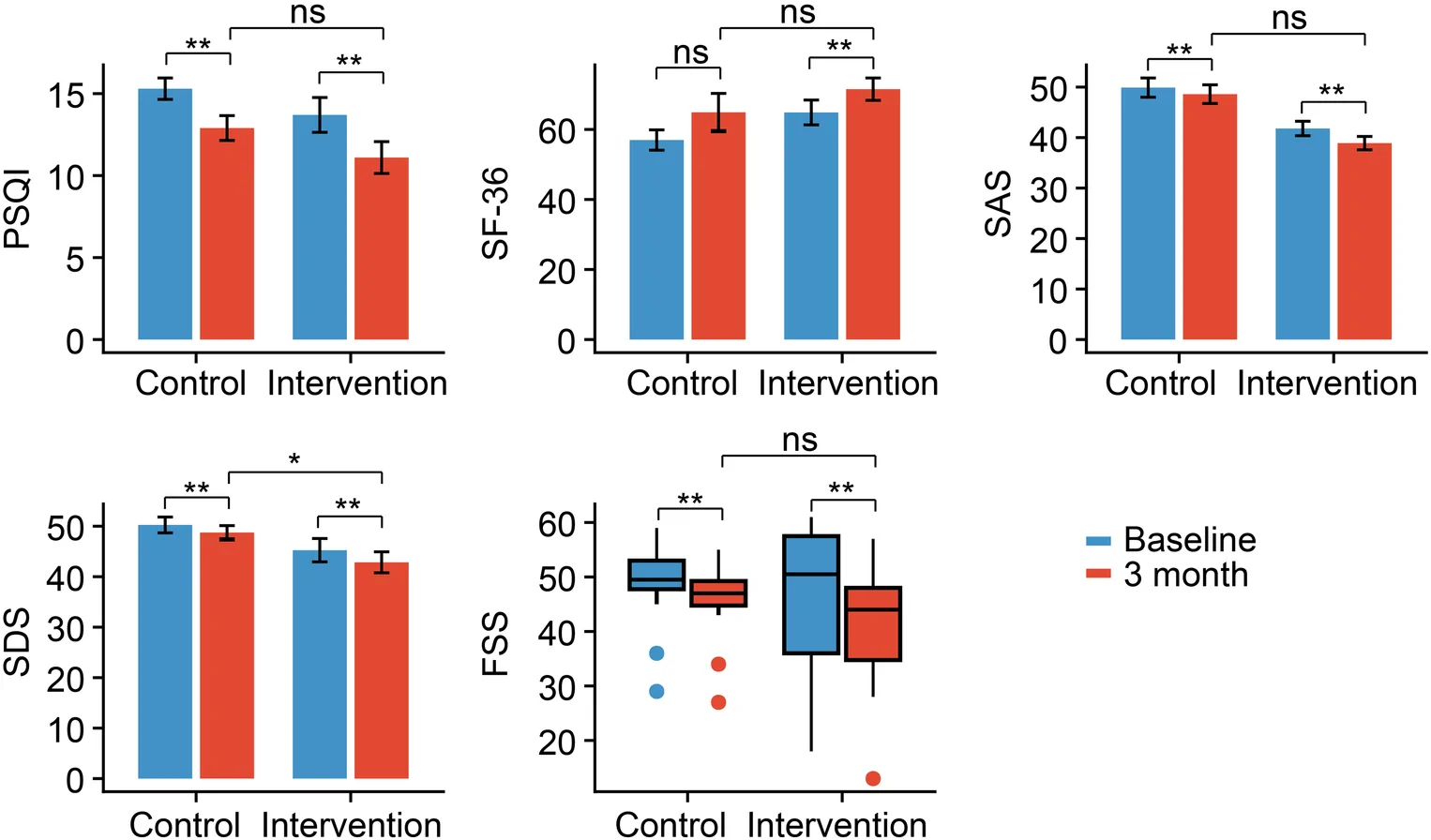
Comparison of scale scores between the control group and intervention group at baseline and 3 months after intervention. PSQI, Pittsburgh sleep quality index; SAS, self-rating anxiety scale; SDS, self-rating depression scale; FSS, fatigue severity scale, SF-36, 36-Item short form health survey; *: 0.001<*P*<0.05; **: *P*<0.001; ns: no significant difference. Normally distributed data are presented using bar charts, while skewed data are displayed via boxplots.

After 3 months of intervention, intra-group and inter-group analyses were conducted on all scale scores. The scores of PSQI, SAS, SDS and FSS were significantly decreased in both groups (all *P* < 0.001). The SF-36 score showed no significant change in the control group (*t* = −1.842, *P* = 0.081), but was significantly increased in the intervention group (*t* = −4.923, *P* < 0.001). Inter-group comparisons showed no significant differences in PSQI, SF-36 and FSS scores between the two groups (all *P* > 0.05). The intervention group had numerically lower SAS (*t* = 4.268, *P* < 0.001) and SDS (*t* = 2.371, *P* = 0.024) scores than the control group (Figure 2).

**Figure 3:**
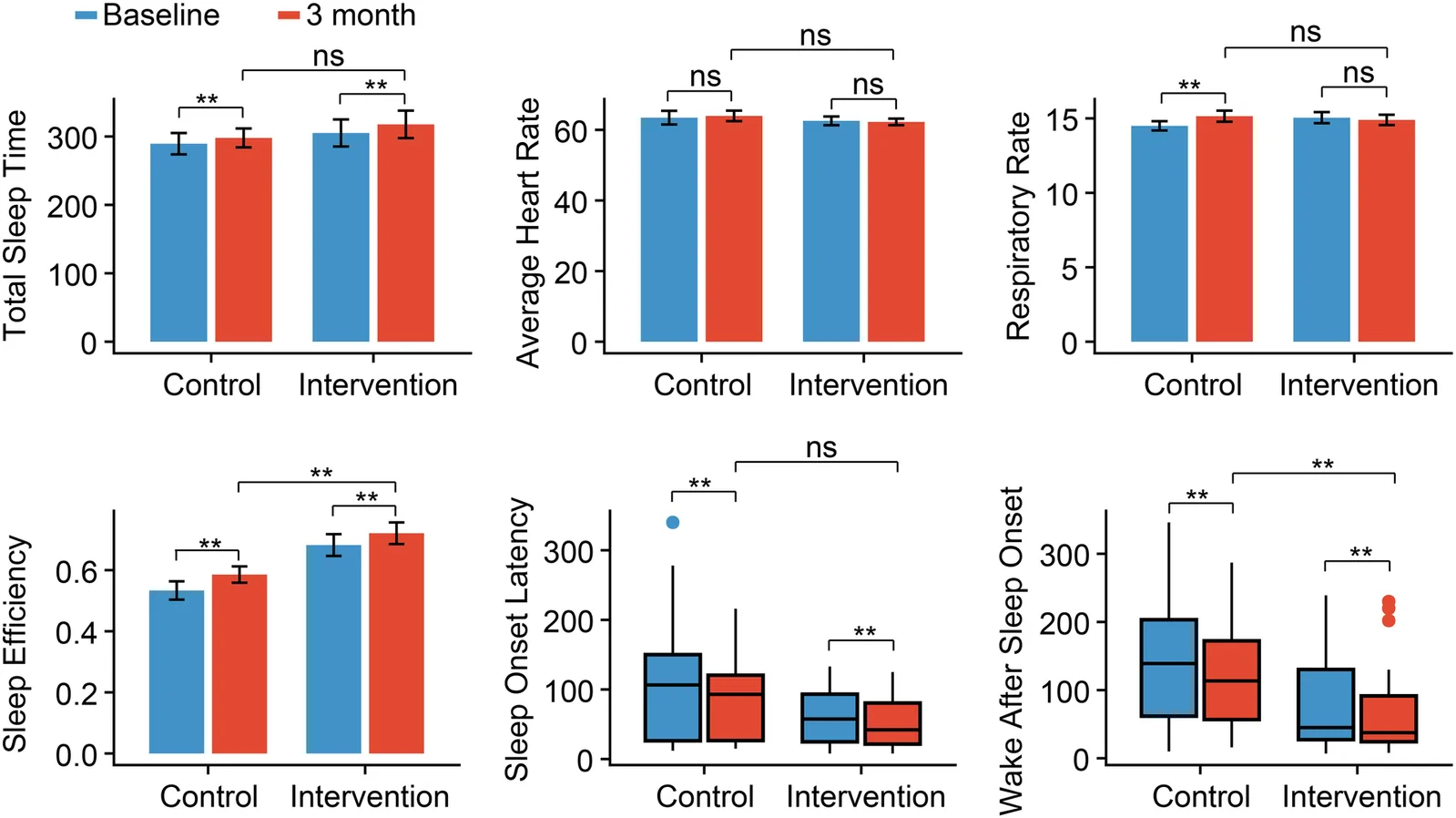
Comparison of objective sleep parameters and nocturnal physiological indicators between the two groups at baseline and 3 months after intervention. **: *P*<0.001; ns: no significant difference. Normally distributed data are presented using bar charts, while skewed data are displayed via boxplots.

For objective indicators, TST, SE, SOL, and WASO all showed statistically significant within-group changes from baseline to the 3-month follow-up in both groups (all *P* < 0.05). The mean respiratory rate was significantly decreased in the control group (*t* = −2.221, *P* = 0.039), while no significant change was found in the intervention group. No significant intra-group differences in mean heart rate were observed in either group (all *P* > 0.05). Inter-group statistical comparisons detected significant differences for SE (*t* = −3.049, *P* = 0.004) and WASO (*U* = 284.5, *P* = 0.023). No significant inter-group differences were found in TST, mean heart rate, mean respiratory rate and SOL (all *P* > 0.05) (Figure 3 and Supplementary Table S7). Sensitivity analysis via analysis of covariance demonstrated that the core intergroup comparative results were stable after adjusting for unbalanced baseline covariates. (Supplementary Table S8).

### 3.3 Inter-group comparisons of indicator changes and change rates after 3-month intervention

**Figure 4:**
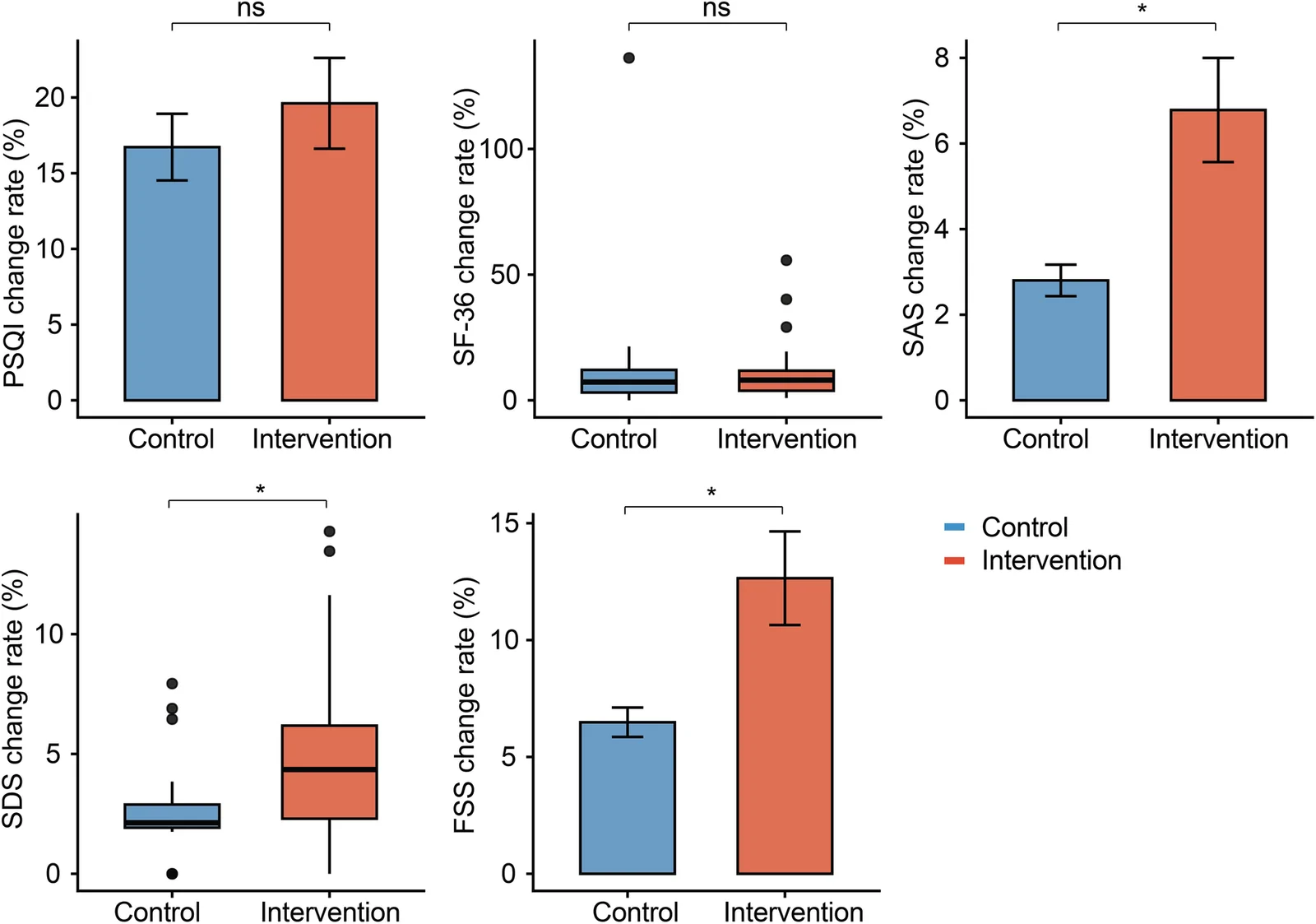
Comparison of percentage change in scale scores between the control group and the intervention group. PSQI: Pittsburgh sleep quality index; SAS: Self-Rating anxiety scale; SDS: Self-Rating depression scale; FSS: Fatigue severity scale; SF-36: 36-Item short form health survey; *: 0.001<*P*<0.05; ns: no significant difference. Normally distributed data are presented using bar charts, while skewed data are displayed via boxplots.

In terms of scale changes after 3 months, no significant inter-group differences were found in the changes of PSQI and SF-36 (all *P* > 0.05). The changes of SAS (*t* = −3.132, *P* = 0.005), SDS (*W* = 126.5, *P* = 0.048) and FSS (*t* = −2.936, *P* = 0.007) in the intervention group were significantly greater than those in the control group (Figure 4).

**Figure 5:**
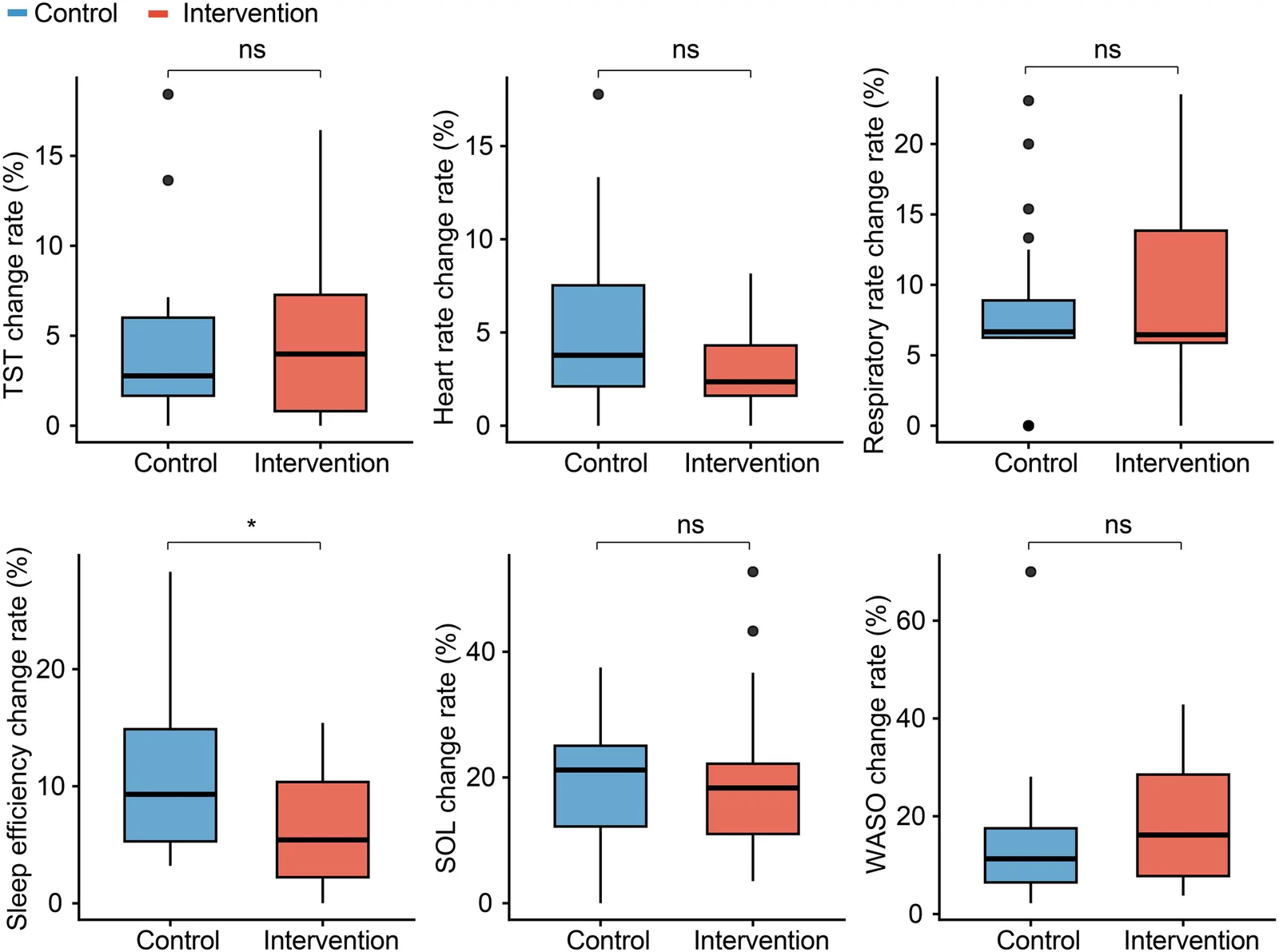
Comparison of percentage change in objective sleep parameters and nocturnal physiological indicators between the two groups. TST, total sleep time; SOL, sleep onset latency; WASO, wake after sleep onset; *, 0.001<*P*<0.05; ns, no significant difference. Normally distributed data are presented using bar charts, while skewed data are displayed via boxplots.

Regarding the change rates of objective sleep and physiological indicators, there were no significant inter-group differences in the change rates of TST (*W* = 189, *P* = 0.776), mean heart rate (*W* = 247.5, *P* = 0.204), mean respiratory rate (*W* = 198, *P* = 0.967) and SOL (*W* = 221.5, *P* = 0.570), as well as WASO (*W* = 148, *P* = 0.164). Only the change rate of SE showed a significant inter-group difference (*W* = 284, *P* = 0.023) (Figure 5).

## 4 Discussion

This pilot study explored preliminary correlations between Resistance Liuzijue Qigong and sleep quality, emotional status, fatigue, and objective sleep parameters among patients with chronic insomnia. Statistical comparisons within this sample revealed larger within-group changes in anxiety and depression scores and greater increases in sleep efficiency values in the intervention arm after 3 months of intervention, alongside a statistically significant inter-group discrepancy in the change rate of sleep efficiency. Importantly, this research adopted a non-randomized open-label design, which may lead to prominent selection and grouping bias and restrict the credibility of observed inter-group differences.

### 4.1 Within-group changes in anxiety and depression scores associated with resistance liuzijue qigong

The present study found that after 3 months of intervention, the SAS and SDS scores of the Resistance Liuzijue Qigong group were numerically lower than those of the control group in inter-group comparison, and the changes of SAS and SDS scores were also larger in the intervention group. This finding suggested a potential additional benefit of Resistance Liuzijue Qigong on comorbid anxiety and depression in patients with chronic insomnia beyond conventional intervention. This result was consistent with existing research findings on mind-body exercises. Systematic reviews have demonstrated that traditional Chinese mind-body exercises can significantly reduce anxiety score (SMD=-1.05) and depression score (SMD=−1.05) in patients with lung cancer, with robust pooled effects (15). Other studies have also pointed out that traditional Chinese mind-body exercises can effectively relieve depression and anxiety in patients with chronic fatigue syndrome (16).

Based on previous literature, several potential mechanisms may be speculated to underlie the observed associations. The first involves the regulatory pathway of rhythmic breathing and vocalization. Traditional Liuzijue Qigong centers on phonation and controlled breathing. The combination of six specific vocalizations and slow exhalation may help stabilize breathing rhythm. Previous studies have suggested that slow breathing combined with vocalization may activate the dACC-PnC neural pathway, which is thought to regulate respiratory and anxiety-related centers in the pons and medulla oblongata, and may thereby contribute to sedative and anxiolytic effects (17). Second, the addition of resistance loads could enhance somatic afferent signaling. Continuous contraction of skeletal muscles may activate muscle mechanoreceptors and transmit proprioceptive information to the cerebral cortex via the spinothalamic tract, potentially inhibiting the overactivation of the medial prefrontal-amygdala pathway implicated in anxiety (18, 19). Third, regular moderate-intensity resistance training has been reported to increase circulating levels of brain-derived neurotrophic factor (BDNF). BDNF is involved in hippocampal neural plasticity and plays a role in limbic emotional regulation networks, which might contribute to alleviating anxiety and depression induced by chronic stress (19).

### 4.2 Optimizing effects of resistance liuzijue qigong on objective sleep parameters

Sleep efficiency refers to the ratio of actual sleep time to time in bed, which is a core objective indicator reflecting sleep continuity. The results showed that the intervention group achieved greater improvements in sleep efficiency and wake after sleep onset, and the inter-group difference in the change rate of sleep efficiency was statistically significant. Previous systematic reviews have verified that traditional Chinese exercises including Tai Chi and Baduanjin can improve actigraphy-measured sleep efficiency in the elderly (20), which is consistent with our findings. Network meta-analysis also revealed that resistance training has prominent advantages in reducing nocturnal awakening, and its efficacy is superior to aerobic exercise and common mind-body exercises (21). Resistance Liuzijue Qigong combines the neural modulation of traditional Qigong and metabolic regulation of resistance training, which may be associated with favorable changes in sleep architecture.

Nevertheless, no significant inter-group differences were found in total sleep time and sleep onset latency. The main reason was that the control group received routine health education rather than blank intervention. Sleep hygiene education itself could produce positive effects on patients’ sleep behaviors. In addition, the baseline physical activity level was different between the two groups, which reduced the sensitivity to detect the additional effects of exercise intervention.

### 4.3 Characteristics of subjective and objective sleep improvements

Although Resistance Liuzijue Qigong was associated with improved objective sleep efficiency and anxiety, no significant inter-group difference was found in total PSQI scores. On the one hand, previous meta-analyses reported that the pooled effect size of Qigong intervention on PSQI was relatively small (SMD=−0.61, 95%*CI*: −1.20, −0.03) (22). Other relevant studies also reported the pooled effect sizes of traditional Chinese exercises on sleep disorders (SMD=−0.48, 95%*CI*: −0.69, −0.27; Hedges's g=−0.60, *P*<0.05) (23, 24). The limited sample size of this study might lead to insufficient statistical power. On the other hand, objective data recorded by actigraphy reflects quantitative changes of sleep continuity, while PSQI is affected by subjective perception and memory bias, so its changes lag behind objective indicators. This suggests that the improvement in objective sleep may not have been fully translated into better subjective sleep experience within the 3-month observation period, and a longer observation period may be needed to detect such changes.

### 4.4 Effects on fatigue and quality of life

Both groups obtained significant reductions in FSS scores after intervention, with no inter-group difference, suggesting that Resistance Liuzijue Qigong and conventional intervention had comparable effects on relieving daytime fatigue in this pilot sample. Relevant systematic reviews have confirmed that traditional Chinese mind-body exercises can alleviate fatigue in patients with chronic fatigue syndrome (16). The improvement of fatigue in this study was mainly attributed to the overall improvement of sleep in both groups, so the additional effect of exercise intervention was not detected. Daytime fatigue in insomnia patients is caused by sleep deprivation, emotional disturbance and physical tension (25–27). The indirect effect of exercise on fatigue may be diluted by routine health education. In terms of quality of life, the SF-36 score was significantly increased in the intervention group but not in the control group, which implied potential benefits of Resistance Liuzijue Qigong. However, the inter-group difference did not reach statistical significance, which needs to be verified by larger sample studies (28, 29).

## 5 Limitations

This study has several limitations. First, this was an open-label controlled study, so expectation bias and assessment bias could not be completely avoided. Participants, trainers and outcome assessors were all aware of group allocation, which may distort self-reported subjective scale outcomes including PSQI, SAS, SDS, FSS and SF-36; although actigraphy-derived objective sleep parameters were less susceptible to bias, unblinded assessors still introduced potential confounding risks (30, 31). Second, the sample size of each group was only 20, resulting in limited statistical power, and some potential inter-group differences might have been missed. *Post-hoc* power analyses further verified that the present sample could only reliably detect large effect sizes above a minimum detectable Cohen's d of 0.909, demonstrating that the current sample size was inadequate to identify small and modest effect sizes, which substantially increased the risk of Type II error. This is consistent with the pilot nature of this study. Third, the intervention period was only 3 months, and long-term follow-up is required to evaluate the sustained effects on subjective sleep and fatigue. Fourth, the unbalanced baseline physical activity levels and baseline anxiety/sleep parameter scores between the two groups constitute major confounding factors. These baseline discrepancies, coupled with the non-randomized design, preclude any causal attribution of the observed between-group differences to the intervention itself. Consequently, our findings can only support exploratory associations, not efficacy in a confirmatory sense. Fifth, the non-randomized design and the different recruitment approaches between groups introduced significant selection and allocation bias, meaning the observed between-group differences should be interpreted as preliminary and hypothesis-generating rather than confirmatory.

## 6 Conclusion

In this prospective open-label non-randomized pilot trial, a 3-month intervention with Resistance Liuzijue Qigong was associated with within-group changes in subjective sleep quality, daytime fatigue, objective sleep efficiency, and sleep continuity in patients with chronic insomnia. Within-group changes (decreases in scores) in anxiety and depression were also observed in the intervention group. However, given the non-randomized design and the pilot nature of this study, these findings should be interpreted as preliminary and hypothesis-generating, and causality cannot be inferred. As a safe and accessible non-pharmacological intervention, Resistance Liuzijue Qigong provides preliminary descriptive evidence for the management of chronic insomnia and lays a foundation for subsequent large-scale randomized controlled trials.

## Data Availability

The raw data supporting the conclusions of this article will be made available by the authors, without undue reservation.
