# Supplementary material for: Associations of resistance liuzijue qigong with sleep and mood in chronic insomnia: a pilot open-label Non-randomized trial
Source: Front Med (Lausanne). 2026 Sep 8;13:1903577. doi: 10.3389/fmed.2026.1903577 (PMC13593511; doi:10.3389/fmed.2026.1903577)
Supplement: Supplementary file 1 [file Table1.docx]

1. Liuzijue: Kinetic and Anatomical Analysis

Liuzijue is a traditional Chinese mind-body exercise that coordinates specific vocalizations ("Xu, He, Hu, Si, Chui, Xi") with breathing and limb movements to promote health. While its benefits in cardiopulmonary rehabilitation and emotional regulation are documented, traditional descriptions lack quantitative analysis of movement kinematics, muscle activation, and respiratory-motor integration. This ambiguity impedes standardized practice and scientific validation. Therefore, this study employs an anatomical and kinesiological framework to analyze each movement component quantitatively, aiming to integrate traditional practice with modern exercise science and facilitate its standardized application.

1.1 Movement Description for "Xu":

Starting Position: Stand with feet parallel, shoulder-width apart. Hands rest at the waist, palms up, little fingers lightly touching the waist. Gaze is downward and forward.

Action: Rotate the torso to the left. Simultaneously, slowly extend the right palm laterally from the waist to approximately shoulder height while exhaling and vocalizing "Xu". The gaze follows the moving hand. Subsequently, retract the right palm and return the torso to the neutral position. The sequence is then repeated on the contralateral side.

List analysis:

| Segment Movement Trends | Section and Joint Movement | Muscle Groups | Prime Mover Muscle | Working Conditions | Nature of Contraction |
| --- | --- | --- | --- | --- | --- |
| The thoracic cage relative to the pelvis | Waist rotation | Trunk rotator muscle group | Abdominal internal and external oblique muscles | Lower fixation | Centripetal contraction |
| The activity shoulder strap relative to the thoracic cage. | Scapula superior rotation | Scapular superior rotation muscle group | Upper and lower fibers of the trapezius muscle and lower fibers of the serratus anterior muscle | Near fixation | Centripetal contraction |
|  | Scapular anterior extension | Scapular anterior extension muscle group | Pectoralis minor, serratus anterior | Near fixation | Centripetal contraction |
| The activity side upper arm relative to shoulder strap | Shoulder flexion | Anterior shoulder flexor group | Anterior deltoid, long head of the biceps brachii, coracobrachialis | Near fixation | Centripetal contraction |
|  | Shoulder adduction | Adductor muscle group | Pectoralis major, latissimus dorsi, teres major | Near fixation | Centripetal contraction |
| Forearm relative to upper arm | Elbow extension | Elbow extensor group | Triceps brachii | Near fixation | Centripetal contraction |

Breathing Guidance for "Xu": The vocalization "Xu" is classified as a dental sound. During exhalation, retract the mouth corners, align the upper and lower incisors with a narrow gap, and position the tongue edges away from the teeth. Inhalation occurs through the nose during the palm retraction phase, while exhalation with the "Xu" sound accompanies palm extension.

Summary: Muscles primarily engaged in the "Xu" technique include the trunk rotators, the upper scapular abductors and anterior elevators, along with the shoulder flexors and adductors. From a traditional perspective, this technique, combined with its specific breathing pattern, is believed to facilitate the regulation of liver qi and the flow of energy through the Shaoyang meridians.

1.2 Movement Description for "He":

Starting Position: Stand with feet parallel, approximately shoulder-width apart. Elbows abducted to shoulder height, palms turned inward with backs touching.

Action: Slowly thrust both palms downward to the navel while exhaling the sound “he”.

List analysis:

| Segment Movement Trends | Section and Joint Movement | Muscle Groups | Prime Mover Muscle | Working Conditions | Nature of Contraction |
| --- | --- | --- | --- | --- | --- |
| Shoulder straps relative to the thoracic cage | Scapular inferior rotation | Scapular inferior rotation muscle group | Pectoralis minor, lower fibers of the trapezius, latissimus dorsi | Near fixation | Centripetal contraction |
| Upper arm relative to shoulder strap | Shoulder adduction | Adductor muscle group | Pectoralis major, latissimus dorsi, teres major | Near fixation | Centripetal contraction |
|  | Shoulder extension | Shoulder extensor group | Latissimus dorsi, posterior deltoid. | Near fixation | Centripetal contraction |
| Forearm relative to upper arm | Elbow extension | Elbow extensor group | Triceps brachii | Near fixation | Centripetal contraction |

Breathing Guidance for "He": The articulation of the "He" sound is linguapalatal. When exhaling, arch the tongue upward and lightly press its sides against the upper incisors. Breath is slowly expelled through the space between the tongue and palate. Inhale through the nose when cupping the palms upward; exhale with the “he” sound when thrusting the palms forward and outward.

Summary: Muscles primarily engaged in the “He” technique include the inferior rotator muscles of the scapular girdle, shoulder adductors and extensors, and elbow extensors. From a traditional Chinese medicine perspective, this technique, combined with its specific respiratory coordination, is theorized to tonify heart qi, purge pathogenic factors from the heart, and contribute to the regulation of cardiac function.

1.3 Movement Description for "Hu":

Starting Position: Stand with feet parallel, approximately shoulder-width apart. Place both palms palm-side inward on either side of the navel.

Action: Open both palms outward, forming a circular arc with the arms. Pronounce the sound “hu” with the mouth.

List analysis:

| Segment Movement Trends | Section and Joint Movement | Muscle Groups | Prime Mover Muscle | Working Conditions | Nature of Contraction |
| --- | --- | --- | --- | --- | --- |
| Shoulder straps relative to the thoracic cage | Scapula superior rotation | Scapular superior rotation muscle group | Upper and lower fibers of the trapezius muscle and lower fibers of the serratus anterior muscle | Near fixation | Centripetal contraction |
| Upper arm relative to shoulder strap | Shoulder flexion | Anterior shoulder flexor group | Anterior deltoid, long head of the biceps brachii, coracobrachialis | Near fixation | Centripetal contraction |
|  | Shoulder abduction | Shoulder abductor muscles | Middle deltoid, supraspinatus muscle | Near fixation | Centripetal contraction |
| Forearm relative to upper arm | Elbow extension | Elbow extensor group | Triceps brachii | Near fixation | Centripetal contraction |

Breathing Guidance for "Hu": The "Hu" sound is classified as a laryngeal articulation. During phonation and exhalation, the sides of the tongue curl upward, the lips are rounded, and the breath stream is directed from the larynx. This creates a centralized column of air within the oral cavity, which is then expelled steadily through the rounded lip aperture.

Summary: The primary muscles involved in performing the “Hu” include the upper rotator muscles of the scapular girdle, along with muscles responsible for shoulder joint flexion and abduction. From a traditional Chinese medicine perspective, this technique, combined with its specific respiratory and vocalization pattern, is theorized to disperse stagnant qi from the spleen and stomach, thereby strengthening spleen function and regulating digestive processes.

1.4 Movement Description for "Si":

Starting posture: Stand with feet parallel, shoulder-width apart. Hold palms at chest height, palms facing forward at shoulder level. Keep elbows down and tucked into ribs. Expand shoulders and chest, tuck head and neck.

Action: Relax shoulders and extend neck. Push palms forward while vocalizing "Si".

List analysis:

| Segment Movement Trends | Section and Joint Movement | Muscle Groups | Prime Mover Muscle | Working Conditions | Nature of Contraction |
| --- | --- | --- | --- | --- | --- |
| Shoulder straps relative to the thoracic cage | Scapular anterior extension | Scapular anterior extension muscle group | Pectoralis minor, serratus anterior | Near fixation | Centripetal contraction |
| Upper arm relative to shoulder strap | Shoulder flexion | Anterior shoulder flexor group | Anterior deltoid, long head of the biceps brachii, coracobrachialis | Near fixation | Centripetal contraction |
| Forearm relative to upper arm | Elbow extension | Elbow extensor group | Triceps brachii | Near fixation | Centripetal contraction |

Breathing Guidance for "Si": The articulation of "Si" is classified as a sibilant dental consonant. During phonation and exhalation, the upper and lower incisors are aligned with a narrow inter-dental gap. The tongue tip lightly contacts the lower dentition, directing the expiratory airflow through the dental aperture. Inhalation occurs nasally during the palm retraction phase, while exhalation coincides with the forward palm thrust.

Summary: Muscles primarily engaged in the “Si” technique include the anterior deltoid muscles of the shoulder girdle, along with those responsible for shoulder flexion and elbow extension. From a traditional Chinese medicine perspective, this technique, combined with its specific respiratory and vocalization pattern, is theorized to tonify lung qi, eliminate pathogenic factors from the lungs, and contribute to the regulation of pulmonary function.

1.5 Movement Description for "Chui":

Starting posture: Stand with feet parallel, about shoulder-width apart. Lightly press palms against the waist points, fingertips angled downward.

Action: Move both palms downward along the waist, sacrum, buttocks, and outer thighs. Slowly squat with both legs while exhaling the sound “chui.”

List analysis:

| Segment Movement Trends | Section and Joint Movement | Muscle Groups | Prime Mover Muscle | Working Conditions | Nature of Contraction |
| --- | --- | --- | --- | --- | --- |
| Shoulder straps relative to the thoracic cage | Scapula superior rotation | Scapular superior rotation muscle group | Upper and lower fibers of the trapezius muscle and lower fibers of the serratus anterior muscle | Near fixation | Centripetal contraction |
| Upper arm relative to shoulder strap | Shoulder flexion | Anterior shoulder flexor group | Anterior deltoid, long head of the biceps brachii, coracobrachialis | Near fixation | Centripetal contraction |
| Forearm relative to upper arm | Elbow extension | Elbow extensor group | Triceps brachii | Near fixation | Centripetal contraction |
| Thigh relative to pelvis | Hip Flexion | Hip extensor muscles | Gluteus maximus | Far fixation | Centrifugal contraction |
| The calf relative to the thigh | Knee flexion | Knee extensor muscles | Quadriceps | Far fixation | Centrifugal contraction |

Breathing Guidance for "Chui": The articulation of "Chui" is classified as a labial sound. During phonation, the tongue is retracted, mouth corners drawn back, and lips protruded then tightened. The expiratory airflow passes through the throat, flows around the tongue laterally, and exits slowly through the constricted lip aperture. Inhalation occurs nasally during palm return to the waist, while exhalation coincides with the descending and encircling palm movement.

Summary: Muscles primarily engaged in the “Chui” technique include the upper rotator muscles of the scapular girdle, along with muscles responsible for shoulder flexion and elbow extension. From a traditional Chinese medicine perspective, this technique, combined with its specific respiratory and vocalization pattern, is theorized to tonify kidney qi, eliminate pathogenic factors from the kidneys, and contribute to the regulation of renal function.

1.6 Movement Description for "Xi":

Starting posture: Stand with feet parallel, about shoulder-width apart. Raise elbows and hands to face, then spread palms outward.

Action: Bend elbows and bring hands back to chest. Slowly press both palms downward to the front of the navel, then spread them outward to the sides of the hips, palms facing outward, while exhaling the “Xi” sound.

List analysis:

| Segment Movement Trends | Section and Joint Movement | Muscle Groups | Prime Mover Muscle | Working Conditions | Nature of Contraction |
| --- | --- | --- | --- | --- | --- |
| Shoulder straps relative to the thoracic cage | Scapular inferior rotation | Scapular inferior rotation muscle group | Pectoralis minor, lower fibers of the trapezius, latissimus dorsi | Near fixation | Centripetal contraction |
| Upper arm relative to shoulder strap | Shoulder extension | Shoulder extensor group | Latissimus dorsi, posterior deltoid. | Near fixation | Centripetal contraction |
|  | Shoulder adduction | Adductor muscle group | Pectoralis major, latissimus dorsi, teres major | Near fixation | Centripetal contraction |
|  | Shoulder internal rotation | Internal rotator muscles | Pectoralis major, latissimus dorsi, teres major | Near fixation | Centripetal contraction |
| Forearm relative to upper arm | Elbow flexion | Elbow Flexor Muscles | Biceps brachii, Brachioradialis, Brachialis | Near fixation | Centripetal contraction |
|  | Elbow extension | Elbow extensor group | Triceps brachii | Near fixation | Centripetal contraction |
|  | Forearm supination | Forearm supinator muscles | Supinator muscle, biceps brachii | Near fixation | Centripetal contraction |

Breathing Guidance for "Xi": The articulation of "Xi" is characterized as a dental fricative. During phonation, the tongue tip maintains light contact with the lower dentition while the mouth corners retract slightly upward. The expiratory airflow is channeled through the inter-dental space with controlled turbulence. Inhalation occurs nasally during upper limb elevation, while exhalation coincides with the pressing and expanding movement phase.

Summary: Muscles primarily engaged in the “Xi” technique include the lower rotator muscles of the shoulder girdle, muscles responsible for shoulder extension, adduction, and internal rotation, as well as muscles involved in elbow flexion, extension, and forearm supination. From the perspective of traditional Chinese medicine theory, this technique, when combined with its specific respiratory and vocalization pattern, is theorized to regulate the Triple Burner (Sanjiao) energy, promote qi circulation among the thoracic, abdominal, and pelvic cavities, and coordinate the physiological functions of the corresponding organ systems.

2. Resistance Training Methods of Liuzijue

This supplement delineates the resistance training protocols for Liuzijue, which integrate elastic band resistance with the core principles of breathing and vocalization to target major muscle groups.

"Xu" Technique Resistance Protocol: Stand with feet parallel, shoulder-width apart. Hold the band with palms facing upward, little fingers lightly resting on your waist. Secure the center of the resistance band around the lower back. Grasp both ends with your hands. Turn your body to the left, slowly extending your right palm through the resistance from your waist toward the left, reaching approximately shoulder height. Simultaneously exhale the “Xu” sound, gaze toward the direction of your extended right palm, then retract the right palm. Return your body to center and repeat the movement on the left side. Throughout the movement, the resistance band applies tension to the upper retraction and anterior extension muscles of the scapular girdle, as well as the muscles responsible for shoulder flexion and adduction (Figure S1a).

"He" Technique Resistance Protocol: Stand with feet parallel, shoulder-width apart. Abduct elbows to shoulder height with palms facing inward, backs of hands touching. Secure the center of the resistance band across the back, grasping both ends. Slowly press palms downward toward the navel against resistance while exhaling with a “he” sound. During this movement, the resistance band engages the downward rotator muscles of the scapular girdle, the adductor and extensor muscles of the shoulder joint, and the elbow extensor muscles (Figure S1b).

"Hu" Technique Resistance Protocol: Stand with feet parallel, shoulder-width apart. Place palms inward on either side of the navel. Secure the center of the elastic band around the lower back. Grasp both ends with your hands, resisting outward pressure with your palms while forming circular arcs with your arms. Exhale while vocalizing the “Hu” sound. Throughout the movement, the elastic band provides resistance to the upper rotator cuff muscles of the scapular girdle and the muscles responsible for shoulder joint flexion and abduction (Figure S1c).

"Si" Technique Resistance Protocol: Stand with feet parallel, shoulder-width apart. Position palms at chest height, facing forward with elbows tucked into ribs. Expand shoulders and chest, tuck head and neck. Secure the center of the resistance band across the back, grasping both ends. Relax shoulders and extend the neck. Push palms forward against resistance while vocalizing the “Si” sound. During this process, the resistance band engages the anterior deltoid muscles, as well as the muscles responsible for shoulder flexion and elbow extension (Figure S1d).

"Chui" Technique Resistance Protocol: Stand with feet parallel, shoulder-width apart. Lightly press palms against the waist points, fingers angled downward. Secure the center of the resistance band across the back, grasping both ends. Move palms downward along the sacral region, buttocks, and outer thighs against resistance while vocalizing the “Chui” sound. During this process, the elastic band provides resistance to the upper rotator muscles of the shoulder girdle, as well as the muscles responsible for shoulder flexion and elbow extension (Figure S1e).

"Xi" Technique Resistance Protocol: Stand with feet parallel, shoulder-width apart. Raise elbows and bring hands to the front. Grasp both ends of the resistance band with palms facing outward against resistance. Bend elbows backward, drawing the band toward the chest. Slowly press palms downward toward the navel, then resist outward to the sides toward the hips, palms facing outward. Exhale the “Xi” sound. Throughout the movement, the resistance band engages the muscles of the scapular belt involved in downward rotation, as well as those responsible for shoulder extension, adduction, abduction, and internal rotation. It also targets the muscles involved in elbow flexion, extension, and forearm supination (Figure S1f).

**Figure S1 Diagram of Liuzijue Resistance Technique**

| **Table S1 Training adherence status of participants in the intervention group** | |
| --- | --- |
| Indicators of training adherence | Values |
| Total prescribed training sessions within 3 months | 900 |
| Mean actual completed training sessions per participant | 834.6 |
| Range of completed training sessions per participant | 771–891 |
| Mean training adherence rate (%) | 92.74 |
| Range of individual adherence rate (%) | 85.67–99.00 |
| Number of participants with adherence rate ≥90% | 16 |
| Number of participants with adherence rate 80%–89.99% | 4 |
| Note: Adherence rate = (Actual completed training sessions / Total prescribed training sessions) × 100%. Total prescribed training sessions were calculated based on 15 min per session, twice daily, 5 days per week for 12 weeks. | |

| **TableS2. Normality Test Results for Quantitative Baseline Characteristics** | | |
| --- | --- | --- |
| Variable | Statistic | *P* |
| Age | 0.957 | 0.129 |
| Body Mass Index | 0.986 | 0.893 |
| Pittsburgh Sleep Quality Index | 0.961 | 0.174 |
| 36-item Short-Form Health Survey | 0.990 | 0.975 |
| Self-Rating Anxiety Scale | 0.957 | 0.132 |
| Self-Rating Depression Scale | 0.974 | 0.476 |
| Fatigue Severity Scale | 0.881 | 0.001 |
| Actigraphy |  |  |
| Total Sleep Time | 0.970 | 0.361 |
| Average Heart Rate | 0.954 | 0.104 |
| Respiratory Rate | 0.891 | 0.001 |
| Sleep Efficiency | 0.961 | 0.180 |
| Sleep Onset Latency | 0.854 | <0.001 |
| Wake After Sleep Onset | 0.898 | 0.002 |
|  | | |

| **Table S3. Normality Test for Outcome Variables at Baseline and 3 Months: Intervention vs Control Groups** | | | | | | |
| --- | --- | --- | --- | --- | --- | --- |
| Outcome Measures |  | Control | |  | Intervention | |
|  |  | baseline | 3 month |  | baseline | 3 month |
| Pittsburgh Sleep Quality Index |  | 0.226 | 0.571 |  | 0.776 | 0.880 |
| 36-item Short-Form Health Survey |  | 0.983 | 0.063 |  | 0.653 | 0.761 |
| Self-Rating Anxiety Scale |  | 0.436 | 0.658 |  | 0.315 | 0.788 |
| Self-Rating Depression Scale |  | 0.904 | 0.795 |  | 0.063 | 0.059 |
| Fatigue Severity Scale |  | 0.005 | 0.002 |  | 0.024 | 0.327 |
| Note: Values above represent P values from normality tests. | | | | | | |

| **Table S4. Normality Test for Actigraphy-derived Variables at Baseline and 3 Months: Intervention versus Control Groups** | | | | | | |
| --- | --- | --- | --- | --- | --- | --- |
| Outcome Measures |  | Control | |  | Intervention | |
|  |  | baseline | 3 month |  | baseline | 3 month |
| Total Sleep Time |  | 0.720 | 0.857 |  | 0.563 | 0.524 |
| Average Heart Rate |  | 0.170 | 0.052 |  | 0.783 | 0.662 |
| Respiratory Rate |  | 0.052 | 0.279 |  | 0.053 | 0.063 |
| Sleep Efficiency |  | 0.134 | 0.294 |  | 0.650 | 0.237 |
| Sleep Onset Latency |  | 0.044 | 0.049 |  | 0.052 | 0.042 |
| Wake After Sleep Onset |  | 0.359 | 0.331 |  | 0.001 | <0.001 |
| Note: Values above represent P values from normality tests. | | | | | | |

| **Table S5. Normality Test Results for Changes in Outcome Measures Before and After Intervention** | | |
| --- | --- | --- |
| Outcome Measures | Control | Intervention |
| Pittsburgh Sleep Quality Index | 0.720 | 0.857 |
| 36-item Short-Form Health Survey | <0.001 | <0.001 |
| Self-Rating Anxiety Scale | 0.240 | 0.071 |
| Self-Rating Depression Scale | <0.001 | 0.028 |
| Fatigue Severity Scale | 0.186 | 0.091 |
| Actigraphy |  |  |
| Total Sleep Time | <0.001 | 0.003 |
| Average Heart Rate | 0.009 | 0.034 |
| Respiratory Rate | 0.015 | 0.054 |
| Sleep Efficiency | 0.031 | 0.122 |
| Sleep Onset Latency | 0.812 | 0.029 |
| Wake After Sleep Onset | <0.001 | 0.058 |
| Note: Values above represent P values from normality tests. | | |

| **Table S6 Post-hoc power analysis for between-group comparisons of primary and secondary outcomes** | | | | | |
| --- | --- | --- | --- | --- | --- |
| Outcome Measures | Statistical test methods | *P* | Observed effect size | Observed statistical power | Minimum detectable Cohen’s d |
| Pittsburgh Sleep Quality Index | Independent samples t-test | 0.207 | 0.406 | 0.240 | 0.909 |
| Self-Rating Anxiety Scale | Independent samples t-test | 0.002 | 1.074 | 0.911 | 0.909 |
| Self-Rating Depression Scale | Independent samples t-test | 0.081 | 0.567 | 0.416 | 0.909 |
| Fatigue Severity Scale | Mann-Whitney U test | 0.786 | 0.045 | 0.059 | 0.909 |
| 36-item Short-Form Health Survey | Independent samples t-test | 0.093 | 0.544 | 0.389 | 0.909 |
| Total Sleep Time | Independent samples t-test | 0.539 | 0.196 | 0.093 | 0.909 |
| Sleep Efficiency | Independent samples t-test | 0.003 | 1.010 | 0.875 | 0.909 |
| Sleep Onset Latency | Mann-Whitney U test | 0.041 | 0.325 | 0.563 | 0.909 |
| Wake After Sleep Onset | Mann-Whitney U test | 0.037 | 0.331 | 0.581 | 0.909 |
| Average Heart Rate | Independent samples t-test | 0.698 | 0.124 | 0.067 | 0.909 |
| Respiratory Rate | Mann-Whitney U test | 0.231 | 0.186 | 0.215 | 0.909 |
| Note: Minimum detectable Cohen’s d = 0.909 was calculated under the setting of n=20 per group, two-tailed α=0.05 and target power=0.8, which remained consistent for all indicators. | | | | | |

| **Table S7 Baseline Characteristics of Enrolled Subjects** | | | | | | | | |
| --- | --- | --- | --- | --- | --- | --- | --- | --- |
| Variables | Intervention group | Control group | Test statistic | *P* | Mean difference | Hodges–Lehmann estimator | 95% *CI* | Cohen’s d |
| General demographic indicators |  |  |  |  |  |  |  |  |
| Age (years, mean ± SD) | 52.30±14.19 | 50.50±12.96 | t = −0.419 | 0.678 | 1.8 | — | [−6.6226, 10.2226] | 0.1325 |
| Gender, n (%) |  |  | χ² = 0.125 | 0.723 | — | — | — | — |
| Male | 5 (25.0) | 6 (30.0) |  |  |  |  |  |  |
| Female | 15 (75.0) | 14 (70.0) |  |  |  |  |  |  |
| BMI (kg/m², mean ± SD) | 23.38±2.42 | 22.84±3.11 | t = −0.618 | 0.54 | 0.545 | — | [−1.1823, 2.2723] | 0.1956 |
| Educational level, n (%) |  |  | χ² = 1.072 | 0.784 | — | — | — | — |
| Primary school and below | 6 (30.0) | 4 (20.0) |  |  |  |  |  |  |
| Junior high school | 7 (35.0) | 10 (50.0) |  |  |  |  |  |  |
| Senior high school | 4 (20.0) | 3 (15.0) |  |  |  |  |  |  |
| College or above | 3 (15.0) | 3 (15.0) |  |  |  |  |  |  |
| Occupation, n (%) |  |  | χ² = 2.692 | 0.26 | — | — | — | — |
| Retired | 8 (40.0) | 5 (25.0) |  |  |  |  |  |  |
| Manual worker/technician/salesperson/farmer | 7 (35.0) | 5 (25.0) |  |  |  |  |  |  |
| Clerk/student/teacher | 5 (25.0) | 10 (50.0) |  |  |  |  |  |  |
| Household registration, n (%) |  |  | χ² = 0.107 | 0.744 | — | — | — | — |
| Urban | 13 (65.0) | 12 (60.0) |  |  |  |  |  |  |
| Rural | 7 (35.0) | 8 (40.0) |  |  |  |  |  |  |
| Physical activity level, n (%) |  |  | — | < 0.001# | — | — | — | — |
| Insufficient activity | 0 (0.0) | 12 (60.0) |  |  |  |  |  |  |
| Moderate activity | 11 (55.0) | 4 (20.0) |  |  |  |  |  |  |
| Vigorous activity | 9 (45.0) | 4 (20.0) |  |  |  |  |  |  |
| Baseline sleep and psychological scales |  |  |  |  |  |  |  |  |
| Pittsburgh Sleep Quality Index (mean ± SD) | 13.70±4.75 | 15.30±2.92 | t = 1.280 | 0.207 | −1.6000 | — | [−4.0431, 0.8431] | 0.4059 |
| 36-Item Short Form Health Survey (mean ± SD) | 64.86±15.86 | 56.98±12.95 | t = −1.720 | 0.093 | 7.879 | — | [−1.0943, 16.8523] | 0.5442 |
| Self-Rating Anxiety Scale (mean ± SD) | 49.90±8.52 | 41.80±6.41 | t = 3.400 | 0.002 | 8.1 | — | [3.4260, 12.7740] | 1.0741 |
| Self-Rating Depression Scale (mean ± SD) | 50.25±7.01 | 45.25±10.33 | t = 1.790 | 0.081 | 5 | — | [−0.4698, 10.4698] | 0.5666 |
| Fatigue Severity Scale, median [IQR] | 50.50 [36.00, 57.50] | 49.50 [47.75, 53.00] | U = 190 | 0.786## | — | 1 | [−8.0000, 6.0000] | — |
| Baseline objective sleep parameters (actigraphy) |  |  |  |  |  |  |  |  |
| Total Sleep Time (min, mean ± SD) | 305.10±88.52 | 289.45±69.84 | t = −0.621 | 0.539 | 15.65 | — | [−33.7690, 65.0690] | 0.1963 |
| Sleep Efficiency (%, mean ± SD) | 0.68±0.16 | 0.53±0.13 | t = −3.190 | 0.003 | 0.1487 | — | [0.0574, 0.2400] | 1.0099 |
| Sleep Onset Latency (min, median [IQR]) | 57.50 [24.75, 93.25] | 106.50 [26.25, 150.00] | U = 276 | 0.041## | — | −43.0000 | [−101.5000, −1.0000] | — |
| Wake After Sleep Onset (min, median [IQR]) | 45.00 [27.25, 130.25] | 139.00 [61.75, 203.25] | U = 278 | 0.037## | — | −51.0000 | [−124.5000, −4.0000] | — |
| Mean heart rate (beats/min, mean ± SD) | 62.55±5.54 | 63.45±8.65 | t = 0.392 | 0.697 | −0.9000 | — | [−5.4032, 3.6032] | 0.1239 |
| Respiratory rate (breaths/min, median [IQR]) | 15 [13, 16.25] | 15 [13, 15.25] | U = 156 | 0.231## | — | 0.5 | [0.0000, 2.0000] | — |
| Normally distributed continuous variables were analyzed by independent-samples t-test and presented as mean ± standard deviation; skewed continuous variables were analyzed by Mann–Whitney U test and presented as median [interquartile range, IQR]. Categorical variables were compared via Pearson χ² test; # Fisher’s exact test; ## Mann–Whitney U test. Mean difference represents the intergroup difference of mean values for normally distributed indicators; Hodges–Lehmann estimator represents the median shift between groups for non-normal skewed indicators. Cohen’s d is the standardized effect size for t-test analyses, which is not applicable for Mann–Whitney U test (marked as —). All confidence intervals were calculated at the 95% level. | | | | | | | | |

| **Table S8 Results of Analysis of Covariance for Sensitivity Analysis After Adjusting Baseline Confounders** | | | |
| --- | --- | --- | --- |
| Outcome Indicator | Adjusted F Statistic | Adjusted P Value | Adjusted R^2^ |
| Post-intervention Pittsburgh Sleep Quality Index | 50.996 | 0.629 | 0.903 |
| Post-intervention Self-Rating Anxiety Scale | 6.659 | 0.0027 | 0.548 |
| Post-intervention Self-Rating Depression Scale | 9.828 | 0.0012 | 0.641 |
| Post-intervention Fatigue Severity Scale | 0.729 | 0.1288 | 0.117 |
| Post-intervention 36-Item Short Form Health Survey | 0.945 | 0.2765 | 0.147 |
| Post-intervention Total Sleep Time | 1.636 | 0.2168 | 0.229 |
| Post-intervention Sleep Efficiency | 24.336 | 0.001 | 0.816 |
| Post-intervention Sleep Onset Latency | 3.085 | 0.1215 | 0.359 |
| Post-intervention Wake After Sleep Onset | 1.173 | 0.6716 | 0.176 |
| Post-intervention Average Heart Rate | 1.296 | 0.062 | 0.191 |
| Post-intervention Average Respiratory Rate | 0.736 | 0.2868 | 0.118 |
| Change value of Self-Rating Anxiety Scale | 38.838 | 0.0027 | 0.876 |
| Change value of Self-Rating Depression Scale | 21.161 | 0.0001 | 0.794 |
| Change value of Fatigue Severity Scale | 1.048 | 0.0395 | 0.16 |
| Change rate of Sleep Efficiency | 12.174 | 0.0006 | 0.689 |
|  | | | |
